# Efficacy of Using Intermittent Theta Burst Stimulation to Treat Negative Symptoms in Patients with Schizophrenia—A Systematic Review and Meta-Analysis

**DOI:** 10.3390/brainsci14010018

**Published:** 2023-12-23

**Authors:** Xiaowei Tan, Shih Ee Goh, Jonathan Jie Lee, Sean David Vanniasingham, Jérôme Brunelin, Jimmy Lee, Phern Chern Tor

**Affiliations:** 1 Department of Mood and Anxiety, Institute of Mental Health, Singapore 539747, Singapore; xiaowei_tan@imh.com.sg (X.T.); shih_ee_goh@imh.com.sg (S.E.G.); jonathan_lee@imh.com.sg (J.J.L.); 2 Department of Addiction Medicine, Institute of Mental Health, Singapore 539747, Singapore; seandavidvan@gmail.com; 3 PSYR2 Team, Lyon Neuroscience Research Center, University Lyon 1, INSERM U1028, CNRS UMR5292, 69000 Lyon, France; jerome.brunelin@ch-le-vinatier.fr; 4 Centre Hospitalier Le Vinatier, 69500 Bron, France; 5 Department of Psychosis, Institute of Mental Health, Singapore 539747, Singapore; jimmy_lee@imh.com.sg; 6 Lee Kong Chian School of Medicine, Nanyang Technological University, Singapore 636921, Singapore; 7 Department of Psychiatric Medicine, Duke-NUS Graduate Medical School, Singapore 169857, Singapore

**Keywords:** iTBS, negative symptoms, schizophrenia, treatment parameters, side effect

## Abstract

Negative symptoms in schizophrenia impose a significant burden with limited effective pharmacological treatment options. Recent trials have shown preliminary evidence for the efficacy of using intermittent theta burst stimulation (iTBS) in treating negative symptoms in schizophrenia. We aim to systematically review the current evidence of iTBS in the treatment of the negative symptoms of schizophrenia as an augmentation therapy. The study protocol was developed and registered on Prospero (registration ID: 323381). MEDLINE, EMBASE, Web of Science (Scopus), PsycINFO and Wan Fang databases were searched for sham-controlled, randomized trials of iTBS among patients with schizophrenia. The mean difference in major outcome assessments for negative symptoms was calculated. The quality of evidence was assessed using the Cochrane Risk of Bias Tool (version 1) and the GRADE system. Moreover, 12 studies including a total of 637 participants were included. Compared to sham treatment, the pooled analysis was in favor of iTBS treatment for negative symptoms (mean weight effect size: 0.59, *p* = 0.03) but not for positive symptoms (mean weight effect size: 0.01, *p* = 0.91) and depressive symptoms (mean weight effect size: 0.35, *p* = 0.16). A significant treatment effect was also observed on the iTBS target site left dorsal prefrontal cortex (mean weight effect size: 0.86, *p* = 0.007) and for stimulation with 80% motor threshold (mean weight effect size: 0.86, *p* = 0.02). Thus, our synthesized data support iTBS as a potential treatment for negative symptoms among patients with schizophrenia. However, the long-term efficacy and safety issues of iTBS in a larger population have yet to be examined.

## 1. Introduction

Negative symptoms are a core component of schizophrenia, associated with a significant diminution or absence of normal behaviors related to motivation and interest (e.g., avolition, anhedonia and social withdrawal) or expression (e.g., blunted effect and alogia), and they account for a large part of the poor functional outcomes, cognitive impairments and long-term morbidity in patients with this disorder [1,2,3]. Negative symptoms in schizophrenia remain a key unmet clinical need with limited pharmacological or psychotherapeutic treatment approaches with small effect sizes and insufficient evidence [4,5]. Therefore, novel treatments for negative symptoms are urgently needed. 

Several non-invasive brain neurostimulation (NIBS) tools have been tested to treat negative symptoms in patients with schizophrenia. Among them, transcranial direct current stimulation (tDCS) was previously found to be effective in ameliorating negative symptoms in patients with schizophrenia. Another type of NIBS, transcranial magnetic stimulation (TMS), is commonly used for conditions like depression, pain and post-acute motor stroke, whereas there is not much evidence regarding its use for schizophrenia symptoms [6,7]. Moreover, published studies and meta-analyses demonstrate that TMS could potentially improve the general functioning of patients with schizophrenia, particularly for positive symptoms like auditory hallucinations [8,9]. The efficacy of the use of the TMS tool to treat negative symptoms in patients with schizophrenia is even less conclusive [10,11,12].

A recent meta-analysis that collected data from 56 studies with 2550 patients provided aggregated evidence that TMS has a small but significant effect size for patients with schizophrenia suffering from negative symptoms [13]. However, there was substantial heterogeneity and a high risk of bias among the included studies, which contained different types of TMS such as rTMS, continuous/intermittent theta burst stimulation (cTBS/iTBS) and deep TMS. It also contained many studies lacking proper sham control. Therefore, a separate and up-to-date analysis of the evidence pertaining to individual forms of TMS, such as TBS, is needed.

TBS involves pulses applied in short bursts (typically three pulses) at a high frequency (50–100 Hz) with an interburst interval in the range of the theta frequency band (3–5 Hz). This has demonstrated powerful effects on synaptic plasticity [14,15]. iTBS is generally considered to facilitate neuronal activity [16], while cTBS is expected to suppress cortical excitability [17]. The iTBS protocol is an extensively researched FDA-approved novel form of magnetic stimulation for the treatment of depression, which can be used to produce the same or greater treatment efficacy for depressive symptoms compared to standard rTMS but in a markedly reduced period of time (3 min of iTBS compared to around 20–38 min of standard rTMS) [18,19,20]. As any TMS protocol that includes more than one session a day is considered an accelerated protocol, there are widely varied protocols of accelerated iTBS described in the literature for patients with depression [18,21,22]. However, the evidence regarding the use of iTBS and accelerated iTBS treatment for patients with schizophrenia remains scarce. In this study, we conduct a systematic review and meta-analysis of the therapeutic efficacy of iTBS (including accelerated iTBS) in the treatment of negative symptoms in schizophrenia vs. sham stimulation.

## 2. Methods

### 2.1. Search Strategy and Eligibility Criteria 

Following the general PRISMA guideline for meta-analysis [23], we searched electronic databases, including MEDLINE (PubMed), Embase, PsychInfo, Web of Science and Wan Fang database (Chinese biomedical database), up to 25 March 2022 with no restriction on publication date, language and length of follow-up. The search terms included “schizophrenia”, “schizoaffective disorder”, “schizophreniform disorder”, “psychotic disorder”, “negative symptom”, “theta burst” and “iTBS”. The detailed search strategy including the analogs used for individual databases is listed in Supplementary Table S1. We manually checked all titles and abstracts of the reference lists after removing the duplicate records with the following inclusion criteria: (1) randomized, sham-controlled trials of iTBS; (2) participants with a primary diagnosis of schizophrenia, schizoaffective disorder, schizophreniform disorder or another psychotic disorder; (3) participant age > 16 years; (4) outcome measured using an established psychometric scale for negative symptoms in schizophrenia such as the negative subscale of the Positive and Negative Syndrome Scale (PANSS-N) [24] or the Scale for Assessment of Negative Symptoms (SANS) [25]. Screening was performed independently by three authors (XWT, JL and TPC), and any disagreements were resolved through consensus. The review protocol was retrospectively registered on PROSPERO (PROSPERO (york.ac.uk), ID: 323381) and was carried out in accordance with “Population, Intervention, comparator and outcomes” (PICO) structure.

### 2.2. Data Extraction and Risk of Bias Assessment 

Original data from the included studies were extracted into pre-defined data extraction forms, which consist of (1) study and patient characteristics; (2) iTBS treatment parameters; (3) follow-up assessment; and (4) iTBS-related side effects and adverse events. If the key data of post-iTBS treatment negative symptoms score were not reported, the corresponding author was contacted via e-mail with a request to provide the data. Otherwise, the paper was excluded from the meta-analysis. 

Risk of bias was independently assessed by three authors at the outcome level using the Cochrane Risk of Bias Tool (version 1) comprising six key domains: (1) random sequence generation (selection bias); (2) allocation concealment (selection bias); (3) blinding of participants (performance bias); (4) blinding of outcome assessment (detection bias); (5) incomplete outcome data (attrition bias); (6) selective reporting (reporting bias). The risk of blinding of personnel (performance bias) was not assessed as it is impractical for clinicians to conduct stimulation treatment while being blinded to the treatment settings. 

Based on the results of the main outcome indicators of the systematic review, the GRADE system [26] was used to evaluate the quality of evidence and grades of recommendation. The quality of evidence grades was as follows: (a) high quality: further research was unlikely to affect the reliability of the efficacy evaluation results; (b) medium quality: further research was likely to affect the reliability of the efficacy evaluation results and was very likely to change the outcome of the evaluation; (c) low quality: further research was very likely to affect the reliability of the efficacy evaluation results and the evaluation outcome was very likely to change; and (d) extremely low quality: the results of any efficacy evaluation were uncertain. The final rating was achieved by consensus from all reviewing co-authors. 

### 2.3. Statistical Methods 

The mean difference (MD) between the iTBS and sham control groups with 95% confidence intervals (CIs) was calculated based on post-treatment scores for each study. If negative symptoms were measured using multiple scales, PANSS-N was chosen for effect size determination. Standardized mean difference (SMD, Cohen’s d) [27] was calculated for pooling of studies with either PANSS-N or SANS scale measurement after iTBS treatment using the inverse variance random effects model in Review Manager 5.4. As suggested by Cohen, SMD values of 0.2–0.5 are considered a small effect size, values of 0.5–0.8 are considered a medium effect size and values > 0.8 are considered a large effect size [28]. Study heterogeneity was assessed using the I^2^ test. Additional sensitivity analyses of pooled effect size were carried out for studies that included a defined severity of prominent negative symptoms in their recruitment of subjects. The definition of prominent negative symptoms differed across studies depending on the assessment scale used (Table 1), i.e., included subjects either had a negative symptom score of at least PANSS-N ≥ 18 or 20 or SANS overall severity ≥ 3. Pooled effect size was also calculated from subgroup studies with stratified iTBS treatment parameters: (1) stimulation site at left dorsolateral prefrontal cortex (L-DLPFC) or cerebellum (including midline cerebellum and left cerebellar vermis); (2) iTBS intensity of 80% motor threshold (MT) or 100% MT. Meta-regression analysis was conducted to examine the moderating effect of iTBS dosage (total number of pulses) on the treatment outcome of post-iTBS negative symptoms. Additional analyses of effect size were conducted for other treatment outcomes, including positive symptoms assessed via positive subscales of PANSS (PANSS-P) or Scale for the Assessment of Positive Symptoms (SAPS) and depressive symptoms assessed via the Calgary Depression Scale (CDSS) or Hamilton Depression Rating Scale (HAMD). If positive symptoms were measured via multiple scales for the same patient, the PANSS-P subscale was chosen for the effect size calculation.

Egger’s funnel plot for assessing publication bias was produced by plotting the standard error of SMD against SMD. Egger’s test was separately conducted by applying linear regression on the normalized effect estimate in each study with the reciprocal of the standard error of the estimate [41]. All statistical analyses and graph plotting were performed with Review Manager 5.4, while the meta-regression analysis, funnel plot and Egger’s test were conducted using Comprehensive Meta-Analysis 3.0 (Englewood, CO, USA).

## 3. Results

### 3.1. Study Design and Patient Characteristics 

**Figure 1:**
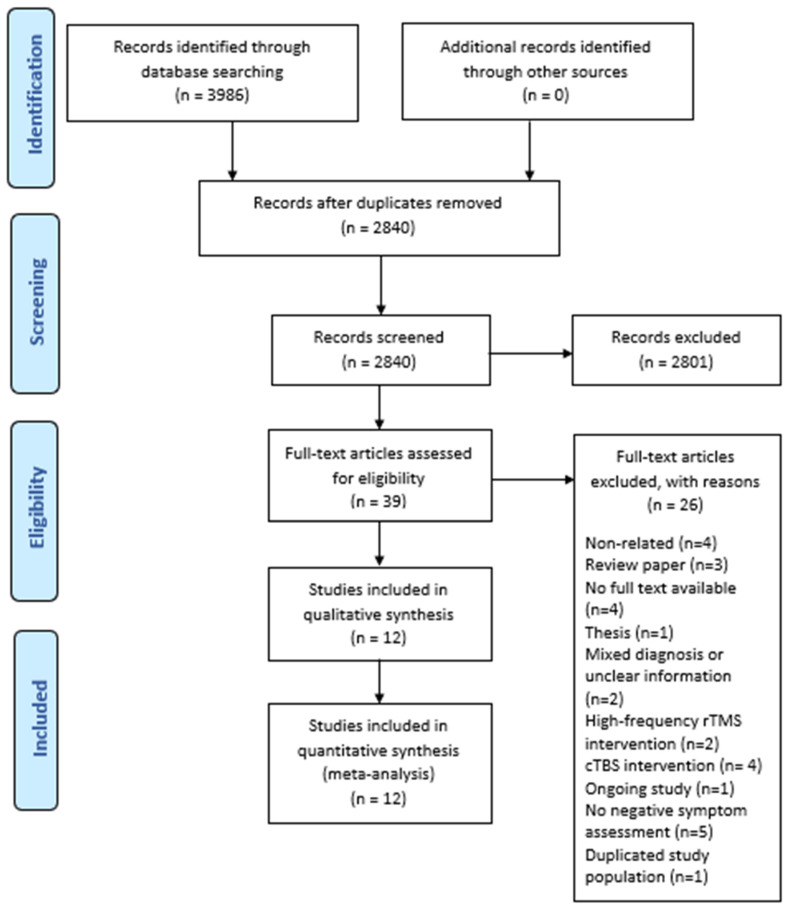
PRISMA flow diagram.

Our initial search strategy identified 3986 papers and reports, which were screened for inclusion. After excluding overlapping reports, non-relevant studies, case reports, conference papers, etc., 12 papers were included for data extraction. These publications include 10 studies with negative symptom outcome data assessed via PANSS-N [30,31,32,33,34,36,37,38,39,40] and 2 studies with negative symptoms assessed via SANS [29,35]. A flow chart describing the article search and selection process is given in Figure 1. 

Study characteristics and patient sociodemographic factors are summarized in Table 1. Most of the studies (8 of 12) recruited only inpatients, 2 studies recruited only outpatients and 2 studies recruited a mix of inpatients and outpatients. Six studies identified patients with prominent negative symptoms before randomization (PANSS-N score ≥ 18 or 20, or SANS overall severity ≥ 3). Most studies (10 of 12) reported that patients and assessors were blinded to treatment group assignment. A total of 637 patients (325 patients in the iTBS group and 312 patients in the sham group) completed the treatment, and their data were included in the analysis.

### 3.2. iTBS Treatment Parameters 

Information regarding the iTBS treatment parameters is listed in Table 2. A total of 9 of the 12 studies used a Magpro TMS machine (Magventure^TM^, Farum, Denmark), with iTBS conducted at either 80% motor threshold (8 of 12) or 100% MT (4 of 12). For each iTBS treatment, most studies used the settings previously reported by Huang et al. [14], i.e., a 50 Hz inner train and 5 Hz inter train (9 of 12). However, the total number of pulses varied from 6000 to 96,000 pulses. The stimulation site was fixed at either the left dorsal lateral prefrontal cortex (L-DLPFC) (9 of 12) or cerebellum (including 1 study targeting the left cerebellar vermis and 2 studies targeting the midline cerebellum). Most studies (11 of 12) reported no change in medication during the treatment phase. 

### 3.3. Treatment Efficacy of iTBS on Negative Symptoms 

**Figure 2:**
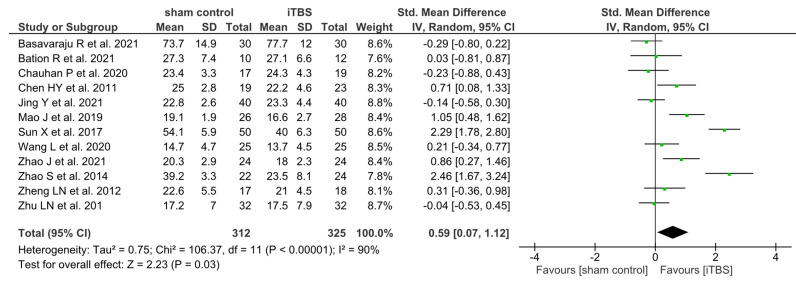
Forest plot of the treatment efficacy of iTBS on negative symptoms [29,30,31,32,33,34,35,36,37,38,39,40].

The pooled effect size of iTBS on negative symptoms from the 12 studies is shown in Figure 2. The mean weighted effect size assessed via SMD was 0.59 (k = 637; −0.07–1.12; *p* = 0.03; I^2^ = 90%), with a significant improvement seen in the active stimulation group as compared to the sham group. 

A sensitivity analysis of the six studies that identified patients with prominent negative symptoms at baseline was performed. The pooled weighted effect size was 1.04 (k = 324; 0.11–1.97; *p* = 0.03; I^2^ = 93%; Table 3).

### 3.4. Subgroup Analysis of the Impact of iTBS Treatment Parameters on Negative Symptoms

The results of the pooled effect sizes stratified via iTBS treatment parameters were assessed (Table 3). The stimulation site showed a significant effect on improvement in negative symptoms at the L-DLPFC site (SMD: 0.86 (k = 477; 0.24, −1.48; *p* = 0.007; I^2^ = 90%)) but not at the cerebellar site (SMD: −0.18 (k = 160; −0.49, 0.13; *p* = 0.27; I^2^ = 0%)). The pooled effect size of studies with 80% MT iTBS intensity was 0.86 (k = 385; 0.15, 1.56; *p* = 0.02; I^2^ = 90%), and there was no significant treatment effect on negative symptoms with 100% MT (SMD: 0.07 (k = 252; −0.38, 0.53; *p* = 0.75; I^2^ = 69%)).

Meta-regression analysis of the 10 studies that reported iTBS dosage showed that the total dosage of iTBS was highly correlated with the effect size of post-treatment negative symptoms (correlation coefficient: 0.027 (0.014, 0.040); *p* < 0.001; Supplementary Figure S1). 

### 3.5. Treatment Efficacy of iTBS on Positive Symptoms and Depressive Symptoms

Additional analyses of the effect of iTBS on positive symptoms and depressive symptoms were conducted. The effect size of iTBS pooled from eight studies with positive symptoms and depressive symptoms assessed is presented in Forest plots (Supplementary Figure S2A and Supplementary Figure S2B, respectively). iTBS stimulation had no significant impact on both positive symptoms (SMD: 0.01 (k = 367; −0.21, 0.23; *p* = 0.91; I^2^ = 9%)) and depressive symptoms (SMD: 0.35 (k = 110; −0.14, 0.84; *p* = 0.16; I^2^ = 39%)) when compared with the sham group.

### 3.6. Dropout Rate and Adverse Events

iTBS treatment had no significant association with the outcome of all-cause dropout rate at the point immediately after treatment (odds ratio: 1.90 (k = 658; 0.77, 4.66; *p* = 0.16; I^2^ = 0%); Supplementary Figure S3).

Of the 12 studies, 5 studies reported a follow-up assessment after iTBS treatment, varying from 2 weeks to 6 months (Supplementary Table S2). Four of these five studies reported a significant time effect of iTBS treatment on the overall improvement in negative symptoms during the follow-up study period. 

iTBS-associated adverse clinical events were reported in 11 of 12 studies (Supplementary Table S3). Headache was the most commonly reported adverse event with 16 (4.9%) of the total 325 patients assigned to the treatment group and 9 (2.9%) of the total 312 patients assigned to the sham control group experiencing it. The other adverse clinical events included dizziness (four patients in the treatment group and one patient in the sham control group) and mania (two patients in the treatment group). 

### 3.7. Quality Assessment of the Included Studies 

The risk of bias for the included studies was low, with the exception of two studies that had an unclear risk of performance bias (the blinding of participants) and detection bias (the blinding of outcome assessment) (Supplementary Figure S4A,B).

**Figure 3:**
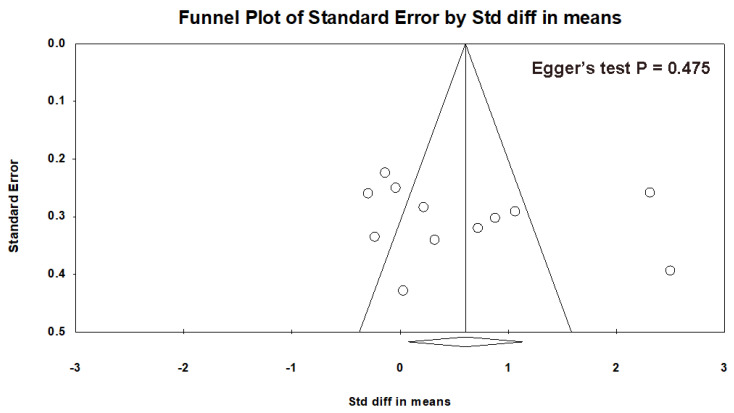
Funnel plot of potential publication bias.

The shape of the funnel plot (Figure 3) resembles an inverted funnel, and the *p*-value of Egger’s test is 0.475, which showed no evidence of publication bias, although there is a lack of publications with large sample sizes.

The overall quality of this meta-analysis evaluating the main outcome of negative symptoms immediately after iTBS treatment was assessed with the GRADE system (Supplementary Table S4). There was an overall low risk of bias for all individual RCT studies. Although the study population was highly heterogeneous (I^2^ = 90%), we performed subgroup analyses and sensitivity analyses to further analyze the effect size within less heterogeneous populations. There was an indirectness of evidence. However, the effect size of negative symptom outcomes was clinically meaningful and the aggregated moderate effect size was dose-dependent. Thus, we believe that the true effect is probably close to the estimated effect reported in our current meta-analysis.

## 4. Discussion

In this systematic review, we found a medium effect size in favor of iTBS efficacy on negative symptoms in schizophrenia compared to sham treatment. Subgroup analysis including only participants with prominent negative symptoms before treatment revealed a larger effect size in favor of iTBS. iTBS settings targeting the L-DLPFC site (vs. cerebellar targets), using 80% MT (vs. 100% MT), and a larger number of total iTBS pulses had larger effects in relieving negative symptoms. A majority of the studies reported a lasting treatment effect on negative symptoms during a follow-up period of 2 weeks or 6 months, with a low incidence of adverse clinical events during the treatment phase. However, there was considerable heterogeneity across the included studies. 

### 4.1. Principal Quantitative Findings 

Several NIBS approaches have been suggested to have promising therapeutic effects on treating negative symptoms in patients with schizophrenia. As mentioned earlier, tDCS is potentially helpful. However, although the FDA assesses tDCS to be safe for adults, there are downsides to consider. For example, tDCS treatment may cause itching, irritation or small burns at the sites of the electrodes [42]. Thus, the overall safety of tDCS is still uncertain as there is a lack of large, long-term studies of tDCS. TBS has recently gained notable attention as a method of regulating cortical excitability in the human brain through modified and patterned rTMS [16,43,44]. The advantage of this stimulation paradigm is that it is able to induce strong and long-lasting effects after a shorter stimulation duration while using a lower stimulation intensity compared to conventional rTMS paradigms. 

Pooled evidence has indicated that iTBS yields moderate to large motor evoked potential (MEP) increments lasting up to 30 min after stimulation, which has been described as facilitating neuronal excitability [45]. A recent trial, the THREE-D study, presented evidence that iTBS is equivalent to the standard left 10 Hz DLPFC rTMS with regard to improvement of depressive symptoms and incidence of side effects [18]. Therefore, iTBS would appear to be an ideal intervention where multiple daily sessions could be applied but in a reduced amount of time. In this meta-analysis, we provided evidence that iTBS has a moderate but significant effect on the negative symptoms of schizophrenia when applied from one to three sessions per day, although this significant effect size may be largely attributed to the results reported by Zhao et al. [38] and Sun et al. [40]. It should also be noted that in most of our selected studies with follow-up assessments, the effect of iTBS on negative symptoms lasted from 2 weeks to 6 months after treatment. These data should be interpreted with caution due to the lack of detailed records of other interventions or adjustments of medications during the follow-up period. Future studies with larger sample sizes will be needed to support our observation of the moderate effectiveness of iTBS on negative symptoms. In fact, there are several currently ongoing clinical trials examining the effect of iTBS on the negative or cognitive symptoms of schizophrenia [46,47]. The data from the impact of iTBS on negative symptoms, either from primary or secondary analysis of those ongoing trials, will contribute to the understanding of iTBS as a therapeutic tool for negative symptoms. 

In psychiatry studies, pseudo-specificity remains a concern when an intervention claims to be effective for certain features of a psychiatric illness. For example, for drug treatment of cognitive dysfunction, which has been intensively studied in patients with depression or schizophrenia, pseudo-specificity is likely to be a major regulatory concern and design challenge, as those cognitive functional improvements may actually be due to the effect of the drug on general cognitive functions or even due to improvements in psychiatric symptoms [48]. A similar issue exists for negative symptoms in schizophrenia. Secondary negative symptoms may be caused by the likes of positive symptoms and depression. In this review, we conducted a subgroup analysis of patients with prominent baseline negative symptoms. The pooled effect size remained significant and increased to a large effect size. An additional analysis of other symptom outcome measures suggested that iTBS seems to have no significant impact on both positive symptoms and depressive symptoms. Thus, our analyses support the adoption of iTBS as a treatment tool specifically for the negative symptoms of schizophrenia. 

Several concerns need to be considered when interpreting the results of iTBS on various domains of psychiatric symptoms. In most of our selected studies, the patients’ positive symptoms had been stabilized via several types of antipsychotics before recruitment and throughout the treatment phase. The pharmacological effects may, thus, mask the effect of iTBS intervention on positive symptoms. Comorbid depressive symptoms, either primarily or secondarily developed, are common in schizophrenia patients [49]. It is not surprising that iTBS may have a large impact on depressive symptoms in schizophrenia patients based on the robust evidence of iTBS as a treatment option with great therapeutic potential for patients with treatment-resistant major depressive disorder [6,18,50]. Thus, the reported insignificant effect size of iTBS on comorbid depressive symptoms in this study may be limited by the relatively low numbers of patients with depressive symptoms assessed (55 patients in the treatment group and 55 patients in the sham group). Future large-scale studies with detailed assessments of comorbid depressive symptoms are needed to clarify the symptom-specific role of iTBS in schizophrenia.

Several subgroup analyses were conducted to examine the potential moderating effect of iTBS stimulation parameters on negative symptoms. Specifically, stimulation at the L-DLPFC was more common (10 of 12 studies) and had superior efficacy (SMD = 0.86) compared to targets at cerebellar sites (SMD = −0.18). These results are in line with an earlier meta-analysis that reported that TMS at the DLPFC site had a superior effect on negative symptoms compared to other target areas [13]. There are increasing amounts of data suggesting that the DLPFC has a privileged relationship with other structures implicated in schizophrenia symptoms, including the midline cerebellum [51]. A recent study also demonstrated that a connectivity breakdown between the cerebellum and DLPFC is associated with negative symptom severity [52]. The network manipulation of the DLPFC in relation to negative symptoms, therefore, will likely be an important area to study when developing stimulation targets in personalized medicine. On the contrary, cerebellar–prefrontal connectivity is established as a biological background underpinning the negative symptoms of schizophrenia [53]. Thus, it is not recommended to exclude the therapeutic potential of the cerebellum as a target site of NIBS treatment for schizophrenia patients [54]. It should be noted that only three studies have investigated the stimulation of the cerebellar sites studied in our analysis. This discrepancy in iTBS treatment outcomes due to different stimulation sites awaits further investigation. 

With regard to stimulation intensity, the majority of the selected studies followed the settings of 80% MT from the original protocol described by Huang et al. [14]. In some other studies, the stimulation was set at 100% MT, which was decided either by the researcher’s clinical experience [55] or with the consideration of the estimated vermis–coil distance [56]. Nevertheless, we observed that iTBS delivered at 80% MT was significantly effective for negative symptoms, which is in contrast to a previous meta-analysis that showed that an rTMS protocol at an intensity of >100% MT may be more effective than other protocols with 80–90% MT (including both traditional 10 Hz/20 Hz rTMS and TBS types of treatment) [57]. The reason for this inconsistency is unclear. The heterogeneous study population included for meta-analysis and the different mechanisms of the magnetic stimulation of iTBS and rTMS may account for this. Moreover, considering the various methods of MT determination in selected RCTs (see Table 2) and the associated variation in efficacy and accuracy [58], it is possible that the difference in stimulation intensity defined by 80% MT vs. 100% MT at an individual level does not reflect the difference in stimulation intensity at the group level. For example, the studies employing 80% MT, which measured MT via visual estimation, may overestimate the actual motor potential, thus resulting in better efficacy than studies employing 100% MT but measuring MT via electromyography (EMG) [59]. However, although EMG does tend to produce lower MTs than visual estimation, it is unlikely that there is a relative difference of 20% MT between these techniques. Therefore, other factors may explain the heightened efficacy of TBS treatment delivered at reduced intensities compared to standard TMS, including the unique pulse timing characteristics of the stimulus. 

Other than stimulation intensity, we also highlighted the large effect size of iTBS on negative symptoms for patients who had been given a larger total number of pulses versus those with a relatively smaller total number of pulses. This implies that iTBS treatment efficacy is dosage-dependent, which is consistent with a previous report that showed a more favorable iTBS treatment effect was observed in patients who received stimulation with ≥1800 pulses per day, for a total of ≥20 sessions in the study [60].

This observation supports the introduction of accelerated iTBS, which can compress more pulses in a shorter period to deliver a stronger effect of brain stimulation, as an alternative to rTMS. However, animal studies provide evidence that although TBS effectively modulates human neocortical excitability, repeated applications of the same TBS protocol at short intervals may not be simply accumulative. It was observed that with repeated iTBS stimulation, some synaptic activity protein markers mainly increased while some somatic neuronal activity markers decreased [61,62]. In a neurophysiology study of iTBS on the DLPFC cortex of healthy volunteers, doubling or tripling the number of iTBS pulses did not result in a stronger potentiation of prefrontal activity [63]. Moreover, in a recent study conducted by Williams et al., who used accelerated, high-dose iTBS to treat patients with treatment-resistant depression, patients’ depressive symptoms remitted very quickly, but the durability of therapeutic response was weak and the relapse rate within the 2-week post-treatment period was very high [50]. Thus, although our analysis supports the utilization of high-dosage iTBS to treat negative symptoms more efficiently, it remains questionable whether the efficacy of high-dosage iTBS stimulation is durable and what the ideal time interval of iTBS delivery for negative symptoms in schizophrenia is. 

Previous studies have reported the long-term efficacy of TMS. A sustained effect during the post-treatment period was reported for iTBS [30], and a delayed effect was reported for high-frequency rTMS [64] in treating the negative symptoms of schizophrenia [30,64]. Similarly, a delayed effect was found while using iTBS to treat depressive symptoms among patients with depression [65]. In our study, mixed results were presented, with the majority of studies reporting a significant effect of iTBS on negative symptoms that lasted from weeks to months after treatment. Patient characteristics, such as age and the duration of illness, may be associated with the duration of iTBS treatment efficacy, but those factors are difficult to examine in our analysis due to the limited sample size and the lack of individual data. As mentioned earlier, the long-term efficacy of iTBS on negative symptoms may also be moderated by other confounders during the follow-up period, such as concurrent medication, social activities and behavior training. Thus, no solid conclusions could be drawn regarding the durability of iTBS efficacy, which is a notable limitation. We suggest future studies include a detailed follow-up measurement. 

In addition to the durability of treatment efficacy, the safety issues of iTBS delivered at high intensities or high dosages remain an issue to be evaluated. TBS has the theoretical potential of conferring an even higher risk of seizure than other types of rTMS protocols because it delivers high-frequency bursts (the most common setting was 50 Hz). There were two cases of mania in our included studies, warranting great caution and the supervised use of iTBS by experienced clinicians. Moreover, mild adverse clinical events, such as headaches, are relatively common (more than 4.9% in the treatment group of our selected RCTs), which is consistent with a previous report of a 5% incidence rate [66]. Headache is also one of the major reasons for withdrawal from iTBS treatment. Rigorous recording of adverse clinical events is suggested for future studies. A detailed treatment guideline should be considered to handle iTBS-associated side effects to encourage the patient’s treatment compliance.

### 4.2. Study Strengths and Limitations

While our results should be interpreted carefully due to the relatively small sample sizes in individual studies, the pooled evidence suggests that iTBS is likely to have a beneficial effect on negative symptoms. Although some of our selected studies were included in previous meta-analyses examining the pooled effect size of TMS or general neurostimulation interventions on the symptoms of schizophrenia [9,12,13,57,67], our current meta-analysis is the first study examining controlled trials of iTBS treatment for negative symptoms in schizophrenia. Assessed via the GRADE system, our analysis is relevant for future clinical practice decisions and policy evaluation. 

The majority of published studies come from Asia. Thus, there is limited external validity of this evidence because the studies are not globally representative of Western or Middle Eastern populations. Our study is also limited by a lack of analysis of potential moderators, such as patients’ age, gender and the duration of illness, of iTBS treatment outcomes. Although the included studies had different inclusion criteria for patient attributes, particularly patient ages during enrolment, these data are overlapping with a large standard deviation. Thus, it is challenging for us to precisely examine the impact of these patient characteristics on treatment outcomes. Nevertheless, a previous study provided evidence that iTBS-associated improvement in negative symptoms among schizophrenia patients showed no correlation with age, age at disease onset and the duration of disease [56]. Future studies specifically examining the impact of patients’ sociodemographic profiles and illness histories on iTBS treatment outcomes are needed. 

### 4.3. Quality Assessment

Our analysis started with high-quality evidence due to the collection of RCTs. The overall risk of individual studies is low except for a lack of blinding methods in several studies. Several patients withdrew from treatment due to adverse clinical events, such as mild headaches. However, mild headaches are common in TMS treatment, and analgesics will help to alleviate this side effect [66], which may or may not have been given to those recruited patients who complained of headaches. In addition, some patients in the sham control group also withdrew from the studies due to headaches. Therefore, iTBS has similar rates of acceptability as a sham. Other than the unclear risks of performance bias and detection bias in some studies, there is high heterogeneity in the study population (I^2^ = 90%), and this heterogeneity remained high in subgroup studies with significant effect sizes (ranging from 90% to 93%). This implies that there are considerable differences between studies in terms of iTBS treatment effects. Unexamined patient characteristics, such as the sociodemographic profile, could be relevant in this regard. However, the observed moderate effect size of iTBS on negative symptoms is linked to considerable clinical improvement measured by the Clinical Global Impressions scale [68]. Thus, our results may be generalizable to a wide population of patients experiencing schizophrenia with negative symptoms.

## 5. Conclusions

In summary, our review and meta-analysis provide evidence of the possible significant efficacy of iTBS on the negative symptoms of schizophrenia. The mean weighted effect size of iTBS is dosage-dependent and highly specific for patients with dominant negative symptoms. Thus, the results of our analyses support the further development of non-invasive iTBS over the frontal cortex, possibly in favor of a high-dosage protocol, as a treatment regimen for negative symptoms. 

## Figures and Tables

**Table 1:** Study characteristics and patient sociodemographic factors.

	Author	Year	Country	Patient Type	Randomization Method	Patient Blind	Assessor Blind	Sham Type	Baseline Selection of Negative Symptoms	Primary Measurement Scale	Total Enrolment	Treatment Assignment	Age, Mean (SD), Year	Education, Mean (SD), Year	Duration of Illness, Mean (SD), Year
1	Basavaraju R et al. [29]	2021	India	Outpatient and inpatient	Computerized algorithm	Yes	Yes	Sham coil	SANS severity ≥ 3	SANS	60	iTBS	31.2 (10.0) ^∆^	11.5 (3.7) ^∆^	8.4 (5.6) ^∆^
												Sham	34.2 (8.1) ^∆^	11.1 (3.9)	10.9 (8.0)
2	Bation R et al. [30]	2021	France	Outpatient	Computerized algorithm	Yes	Yes	Sham coil	PANSS-N ≥ 20, 2 items ≥ 4	PANSS-N	22	iTBS	42.3 (9.4)	11.5 (2.5)	15.0 (5.9)
												Sham	41.6 (12.6)	12.1 (2.8)	17.1 (15.4)
3	Chauhan P et al. [31]	2020	India	Inpatient	Block randomization	Yes	Yes	Sham coil	NA	PANSS-N	36	iTBS	41.7 (8.9)	~8.3 *	16.1 (5.5)
												Sham	39.4 (8.2)	~9.6 *	13.0 (7.0)
4	Chen HY et al. [32]	2011	China	Inpatient	Computerized algorithm	Yes	Yes	Similar sound	PANSS-N ≥ 20	PANSS-N	46	iTBS	37.4 (11.8)	12.0 (2.2)	NA
												Sham	39.7 (13.3)	11.0 (2.6)	NA
5	Jin Y et al. [33]	2021	China	Inpatient	Computerized algorithm	Yes	Yes	180°	NA	PANSS-N	80	iTBS	48.7 (9.7)	7.2 (2.5)	8.9 (4.0)
												Sham	47.8 (10.6)	6.4 (2.4)	8.4 (4.3)
6	Mao J et al. [34]	2019	China	Inpatient	NA	NA	NA	NA	PANSS-N ≥ 18	PANSS-N	60	iTBS	52.8 (7.1)	9.1 (1.3)	27.7 (9.2)
												Sham	53.5 (5.5)	9.3 (1.8)	27.5 (9.9)
7	Sun X et al. [35]	2018	China	Inpatient	NA	Yes	Yes	No treatment	PANSS-N ≥ 20, lasted for at least 6 weeks	SANS	100	iTBS	51.2 (11.4)	NA	NA
												Sham	50.9 (12.1)	NA	NA
8	Wang L et al. [36]	2020	China	Outpatient	Coin toss	Yes	Yes	Sham coil	NA	PANSS-N	58	iTBS	24.0 (4.4)	12.1 (2.6)	5.1 (3.8)
												Sham	26.6 (9.0)	12.1 (2.7)	4.9 (5.3)
9	Zhao J et al. [37]	2021	China	Inpatient	Random number table	NA	NA	90°	NA	PANSS-N	52	iTBS	62.5 (3. 3)	9.3 (2.3)	31.3 (8.9)
												Sham	64.0 (3. 6)	9.5 (2.4)	35.0 (8.9)
10	Zhao S et al. [38]	2014	China	Outpatient and inpatient	Random number table	Yes	Yes	180°	PANSS-N ≥ 20, with at least one of the negative symptom scores > 3	PANSS-N	48	iTBS	47.7 (11.8)	12.9 (0.9)	NA
												Sham	46.7 (13.1)	13.8 (0.1)	NA
11	Zheng LN et al. [39]	2012	China	Inpatient	Computerized algorithm	Yes	Yes	180°	NA	PANSS-N	39	iTBS	55.6 (5.8)	~10.2 *	32.9 (8.1)
												Sham	56.4 (9.3)	~9.6 *	31.7 (7.2)
12	Zhu L et al. [40]	2021	China	Inpatient	Odd–even number sequence	Yes	Yes	180°/90°	NA	PANSS-N	64	iTBS	35.2 (7.1) ^∆^	10.9 (3.2)	15.4 (7.8)
												Sham	35.3 (6.1) ^∆^	10.2 (3.9)	15.8 (6.5)

Abbreviations: PANSS-N—the Positive and Negative Syndrome Scale–negative symptoms subscale; SANS—the Scale for the Assessment of Negative Symptoms; iTBS—intermittent theta burst stimulation; SD—standard deviation; NA—no data or not described. ^∆^ data from total enrolled patients before treatment. * converted from categorical education grade.

**Table 2:** iTBS treatment parameters.

	Author	TMS Machine	MT Method	Target Site	Intensity	iTBS Sessions/Day	Inner Train Frequency (Hz)	Inter Train Frequency (Hz)	Inter Train Interval (s)	Total Number of Sessions	Number of Pulses/Sessions	Total Number of Pulses	Concurrent Antipsychotics
1	Basavaraju R et al. [29]	MagPro X100	Rossini–Rothwell MEP	Midline cerebellum	100% MT	2	50	5	8	10	600	6000	No change in medication
2	Bation R et al. [30]	MagPro X100	Visual observation	L-DLPFC	80% MT	2	50	5	8	20	990	19,800	No change in medication
3	Chauhan P et al. [31]	MagPro-R30	Rossini–Rothwell MEP	Midline cerebellum (cerebellar)	80% MT	2	50	5	8	10	600	6000	No change in medication
4	Chen HY et al. [32]	MagPro X100	NA	L-DLPFC	80% MT	1	50	NA	NA	20	2400	48,000	No change in medication
5	Jin Y et al. [33]	JunJiang RT-100	Visual observation	L-DLPFC	100% MT	1	NA	NA	NA	20	NA	NA	No change in medication
6	Mao J et al. [34]	MagPro R100	NA	L-DLPFC	80% MT	1	NA	NA	NA	20	NA	NA	No change in medication
7	Sun X et al. [35]	NA	NA	L-DLPFC	80% MT	1	NA	5	120	40	2400	96,000	NA
8	Wang L et al. [36]	MagStim Rapid2	Sequential testing MEP	L-DLPFC	80% MT	3	50	5	8	42	600	25,200	No change in medication
9	Zhao J et al. [37]	NA	NA	L-DLPFC	100% MT	1	50	5	NA	20	600	12,000	No change in medication
10	Zhao S et al. [38]	MagPro X100	Rossini–Rothwell MEP	L-DLPFC	80% MT	1	50	5	NA	20	2400	48,000	No change in medication
11	Zheng LN et al. [39]	MagPro X100	NA	L-DLPFC	80% MT	1	50	5	8	5	1200	6000	No change in medication
12	Zhu LN et al. [40]	MagPro X100/CCY-I	NA	Midline cerebellum (cerebellar)	100% MT	1	50	5	8	10	600	6000	No change in medication

Abbreviations: MEP—motor evoked potential; L-DLPFC—left dorsolateral prefrontal cortex; MT—motor threshold; NA—no data or not described.

**Table 3:** Subgroup analysis of iTBS treatment efficacy on negative symptoms.

	Subgroup Attributes	Pooled Effect Size				Total Number of Patients
		SMD	95% CI	*p*-Value	I^2^	
1	Studies with baseline prominent negative symptoms	1.04	0.11, 1.97	0.030	93%	324
2	Stimulation site: L-DLPFC	0.86	0.24, 1.48	0.007	90%	477
	Stimulation site: cerebellum	−0.18	−0.49, 0.13	0.270	0%	160
3	80% MT	0.86	0.15, 1.56	0.020	90%	385
	100% MT	0.07	−0.38, 0.53	0.750	69%	252

Abbreviations: L-DLPFC: left dorsolateral prefrontal cortex; MT: motor threshold; SMD: standard mean difference; CI: confidence interval.

## Data Availability

Meta-analysis and quality assessment data are available from the corresponding author, P.C.T., upon reasonable request. The data are not publicly available due to unpublished data were provided by cited authors for meta-analysis.

## Supplementary Materials

The following supporting information can be downloaded at: https://www.mdpi.com/article/10.3390/brainsci14010018/s1, Figure S1: Meta-regression plot of post-treatment effect size of negative symptoms (standard difference in means) on iTBS total dosage; Figure S2: Forest plot of the treatment efficacy of iTBS on positive symptoms (A) and (B) depressive symptoms; Figure S3: Forest plot of the treatment efficacy of iTBS on all-cause dropout rate immediately after treatment; Figure S4: Risk of bias assessment (A) and individual study (B); Table S1: Literature searching strategies; Table S2: Follow-up assessment of negative symptoms; Table S3: Safety and adverse events of iTBS treatment for patients with schizophrenia; Table S4: *Grading* of Recommendations, *Assessment*, Development and Evaluations (GRADE) form.
