# Supplementary material for: Efficacy of Using Intermittent Theta Burst Stimulation to Treat Negative Symptoms in Patients with Schizophrenia—A Systematic Review and Meta-Analysis
Source: Brain Sci. 2023 Dec 23;14(1):18. doi: 10.3390/brainsci14010018 (PMC10813174; doi:10.3390/brainsci14010018)
Supplement: Supplementary file 1 [file brainsci-14-00018-s001.zip › brainsci-2730783-supplementary.pdf]

Supplementary materials for publication

1. Supplementary Table S1: Literature searching strategies
2. Supplementary Figure S1: Meta-regression plot of post-treatment effect size of negative symptoms (standard difference in means) on iTBS total dosage
3. Supplementary Figure S2: Forest plot of the treatment efficacy of iTBS on positive symptoms (A) and Depressive (B) symptoms
4. Supplementary Figure S3: Forest plot of the treatment efficacy of iTBS on all-cause dropout rate at immediately after treatment
5. Supplementary Table S2: Follow-up assessment of negative symptoms
6. Supplementary Table S3: Safety and adverse events of iTBS treatment for patients with schizophrenia
7. Supplementary Figure S4: Risk of bias assessment (A) and individual study (B)
   Supplementary Figure 3B: Risk of bias assessment of individual study
8. Supplementary Table S4: GRADE (Grading of Recommendations, Assessment, Development and Evaluations) form

1. Supplementary Table S1: Literature searching strategies

No.	Database name	Searching terms	Searching date	Total hits
1	Medline	“schizophreni*” OR "schizoaffective disorder" OR "schizophreniform disorder" OR "schizophrenia"[MeSH Terms] OR “negative symptom*” OR "Psychotic Disorders"[MeSH Terms] OR "Psychotic Disorder*") AND "theta burst" OR "iTBS"	3/25/2022	640
2	Wan fang DATA (wanfangdata.com.cn)	“theta burst stimulation” or “iTBS” and “negative symptoms” and “schizophrenia”	3/25/2022	681
3	EMBASE	schizophrenia*':ab,kw,ti OR 'schizoaffective disorder':ab,ti,kw OR 'schizophreniform disorder':ab,kw,ti OR 'schizophrenia spectrum disorder'/exp OR 'psychotic disorder*':ab,kw,ti OR ‘negative symptom*’:ab,kw,ti) AND ('theta':ab,kw,ti OR 'itbs':ab,kw) AND ('article'/it OR 'article in press'/it OR 'review'/it	3/25/2022	1139
4	PsycINFO	schizophrenia* OR "schizoaffective disorder" OR "schizophreniform disorder" OR "Psychotic Disorder*" OR “negative symptom*”) AND "theta" OR "iTBS"	3/25/2022	529
5	Web of Science (Scopus)	schizophreni* OR "schizoaffective disorder" OR "schizophreniform disorder" OR "Psychotic Disorder*" OR “negative symptom*”) AND ("theta" OR "iTBS")	3/25/2022	997

2. Supplementary Figure S1: Meta-regression plot of post-treatment effect size of negative symptoms (standard difference in means) on iTBS total dosage

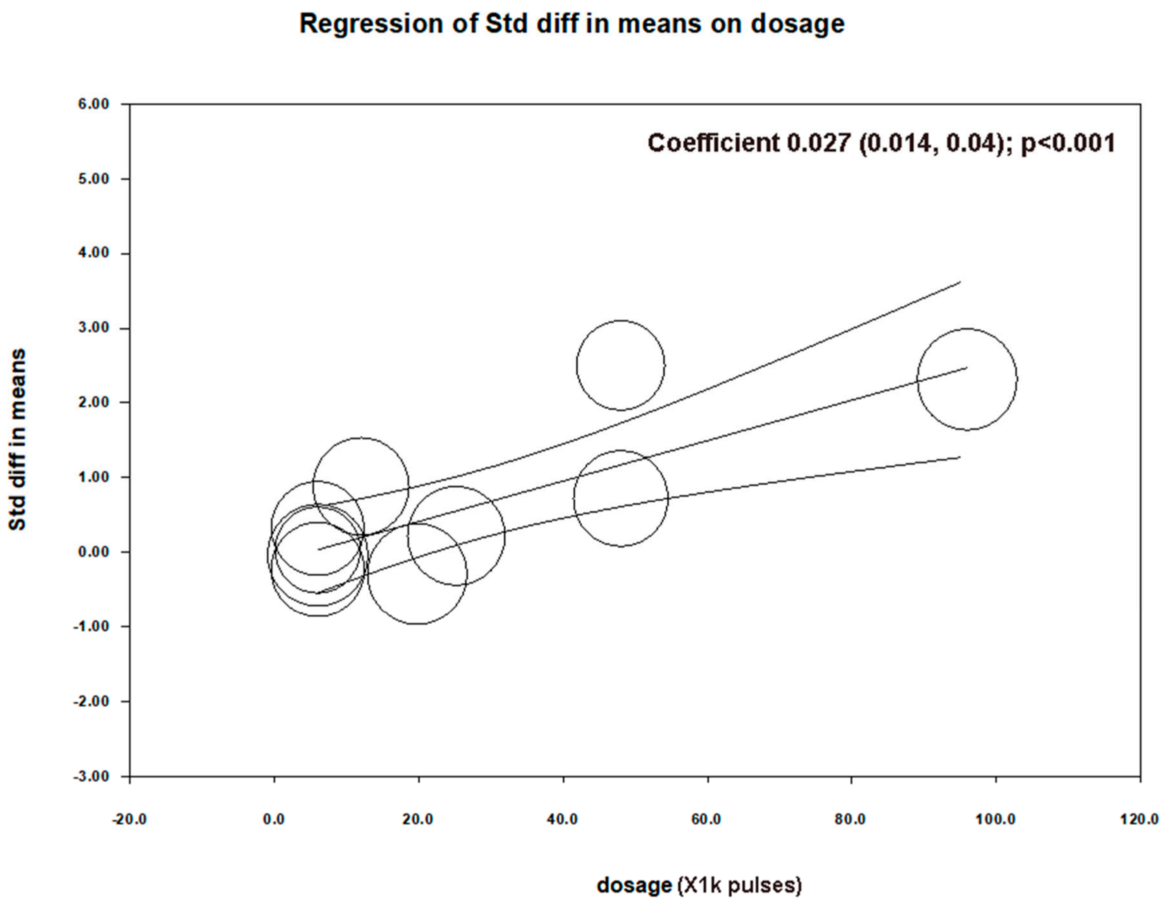

3. Supplementary Figure S2 Forest plot of the treatment efficacy of iTBS on positive symptoms (**A**) and depressive symptoms (**B**)

(**A**)

	sham control			iTBS				Std. Mean Difference
**Study or Subgroup**	**Mean**	**SD**	**Total**	**Mean**	**SD**	**Total**	**Weight**	**IV, Random, 95% CI**
Bation R et al. 2021	9.2	2.5	10	10	2.6	12	6.3%	-0.30 [-1.15, 0.54]
Chauhan P et al. 2020	17.7	1.9	17	18.9	3	19	9.9%	-0.46 [-1.13, 0.20]
Chen HY et al. 2011	12.1	2.8	19	11.6	2.7	23	11.7%	0.18 [-0.43, 0.79]
Jing Y et al. 2021	12.8	2.6	40	13.1	2	40	20.8%	-0.13 [-0.57, 0.31]
Mao J et al. 2019	13	3.4	26	13.7	4.9	28	14.7%	-0.16 [-0.70, 0.37]
Wang L et al. 2020	13.3	6.6	25	10.8	3.3	25	13.5%	0.47 [-0.09, 1.03]
Zhao J et al. 2021	9.3	2.2	24	8.5	2	24	13.1%	0.37 [-0.20, 0.95]
Zheng LN et al. 2012	12.8	4.9	17	13.1	5.6	18	10.0%	-0.06 [-0.72, 0.61]
**Total (95% CI)**			**178**			**189**	**100.0%**	**0.01 [-0.21, 0.23]**

Heterogeneity: Tau² = 0.01; Chi² = 7.72, df = 7 (P = 0.36); I² = 9%
Test for overall effect: Z = 0.11 (P = 0.91)

Std. Mean Difference
IV, Random, 95% CI

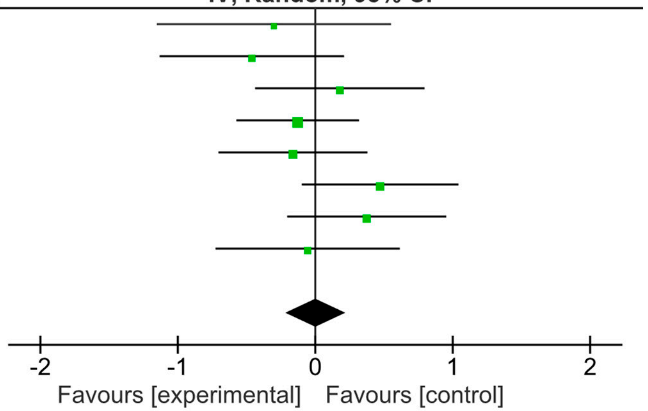

(**B**)

	sham control			iTBS				Std. Mean Difference
**Study or Subgroup**	**Mean**	**SD**	**Total**	**Mean**	**SD**	**Total**	**Weight**	**IV, Random, 95% CI**
Basavaraju R et al. 2021	0.6	2.7	30	0.3	2.4	30	53.5%	0.12 [-0.39, 0.62]
Wang L et al. 2020	5.4	3.3	25	3.8	1.5	25	46.5%	0.61 [0.05, 1.18]
**Total (95% CI)**			**55**			**55**	**100.0%**	**0.35 [-0.14, 0.84]**

Heterogeneity: Tau² = 0.05; Chi² = 1.65, df = 1 (P = 0.20); I² = 39%
Test for overall effect: Z = 1.40 (P = 0.16)

Std. Mean Difference
IV, Random, 95% CI

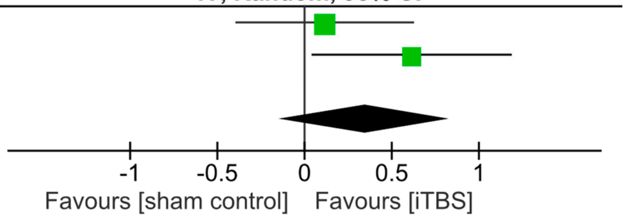

4. Supplementary Figure S3: Forest plot of the treatment efficacy of iTBS on all-cause dropout rate

	sham control		iTBS			Odds Ratio
**Study or Subgroup**	**Events**	**Total**	**Events**	**Total**	**Weight**	**M-H, Random, 95% CI**
Basavaraju R et al	0	30	0	30		Not estimable
Bation R et al	0	10	0	12		Not estimable
Chauhan P et al	1	17	1	19	9.9%	1.13 [0.06, 19.50]
Chen HY et al	3	22	1	24	14.7%	3.63 [0.35, 37.83]
Jin Y et al	0	40	0	40		Not estimable
Mao J et al	4	30	2	30	25.5%	2.15 [0.36, 12.76]
Sun X et al	0	50	0	50		Not estimable
Wang L et al	0	25	0	25		Not estimable
Zhao J et al	2	26	2	26	19.4%	1.00 [0.13, 7.69]
Zhao S et al	2	24	0	24	8.4%	5.44 [0.25, 119.63]
Zheng LN et al	3	20	2	20	22.1%	1.59 [0.24, 10.70]
Zhu LN et al	0	32	0	32		Not estimable
**Total (95% CI)**		**326**		**332**	**100.0%**	**1.90 [0.77, 4.66]**
Total events	15		8			

Heterogeneity: Tau² = 0.00; Chi² = 1.31, df = 5 (P = 0.93); I² = 0%

Test for overall effect: Z = 1.40 (P = 0.16)

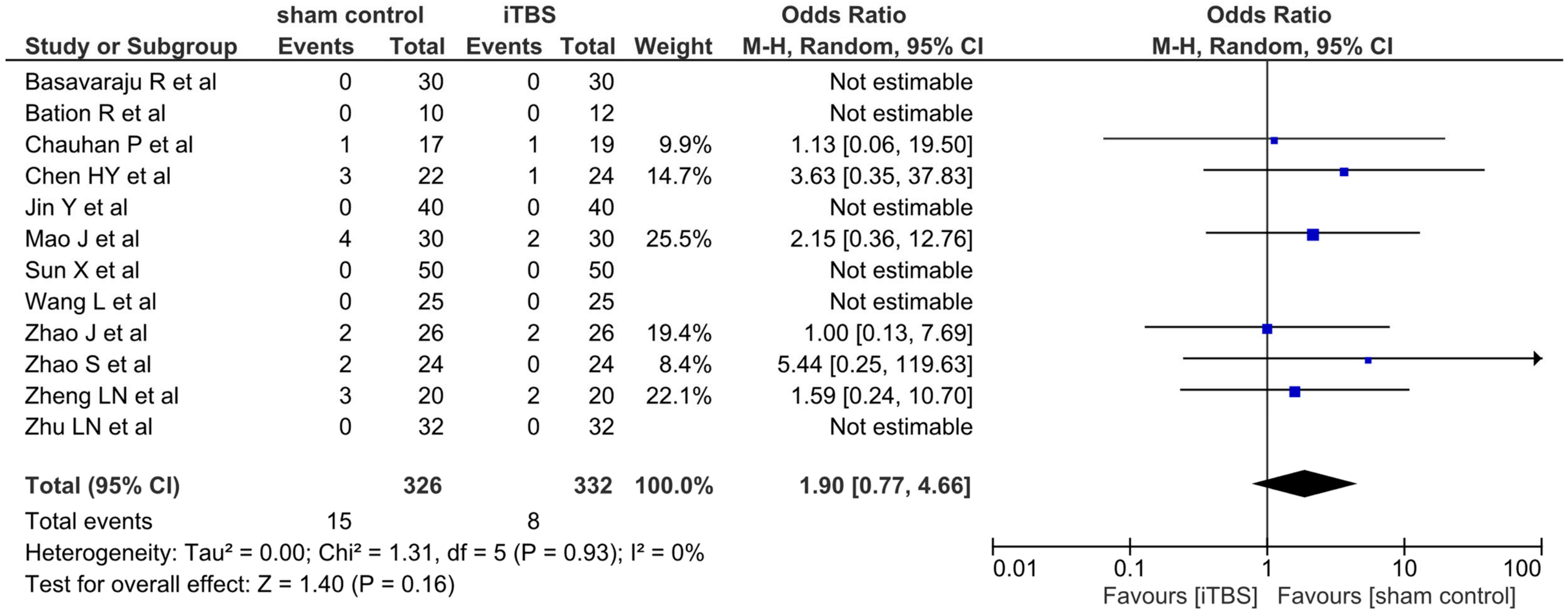

5. Supplementary Table S2: Follow-up assessment of negative symptoms

No	Author	Follow-up period	Treatment assignment	Follow-up score	Significant time effect within study period (Mixed Models Repeated Measures)
1	Basavaraju R et al	5 weeks	iTBS	76.4 (2.7) *	Yes
			sham	72.0 (3.0) *	
2	Bation R et al	6 months	iTBS	Unclear *†	Yes
			sham	Unclear *†	
3	Chauhan P et al	2 weeks	iTBS	23.1 (4.6)	No
			sham	21.8 (3.2)	
4	Chen HY et al	no	iTBS	_	_
			sham	_	_
5	Jin Y et al	no	iTBS	_	_
			sham	_	_
6	Mao J et al	no	iTBS	_	_
			sham	_	_
7	Sun X et al	no	iTBS	_	_
			sham	_	_
8	Wang L et al	2 months	iTBS	12.08 (3.66)	Yes
			sham	14.52 (4.78)	
9	Zhao J et al	no	iTBS	_	_
			sham	_	_
10	Zhao S et al	no	iTBS	_	_
			sham	_	_
11	Zheng LN et al	no	iTBS	_	_
			sham	_	_
12	Zhu L et al	6 months	iTBS	14.5 (6.1)	Yes
			sham	17.2 (6.7)	

* assessed by The Scale for the Assessment of Negative Symptoms (SANS)

† data was displayed in graph

6. Supplementary Table S3: Safety and adverse events of iTBS treatment for patients with schizophrenia

No	Author	Adverse events during treatment phase		Adverse events during follow-up period	
		iTBS	sham	iTBS	sham
1	Basavaraju R et al	2 patient mania/hypomania and 1 patient neck pain	No	No	No
2	Bation R et al	mild headache*	mild headache*	No	2 patient an exacerbation of positive symptoms
3	Chauhan P et al	5 patient mild headache	2 patient mild headache	NA	NA
4	Chen HY et al	4 patient headaches	No	_	_
5	Jin Y et al	1 patient dizzy、1 patient prickling pain、2 patients scalp tingling	1 patient headache、1 patient dizzy, 1 patient scalp tingling	_	_
6	Mao J et al	7 patient headache, 1 patient sleepless	5 patient headaches	_	_
7	Sun X et al	3 patient mild headache*			
8	Wang L et al	No	No	No	No
9	Zhao J et al	1 patient cannot tolerate treatment	No	_	_
10	Zhao S et al	2 patient insomnia	1 patient severe headache	_	_
11	Zheng LN et al	No	NA	_	_
12	Zhu LN et al	3 patient mild dizziness, pain, nausea and other symptom	No	No	No

Abbreviations: iTBS, intermittent theta burst stimulation; NA, no data or not described

7. Supplementary Figure S4A: Risk of bias assessment (**A**) and individual study (**B**)

(**A**)

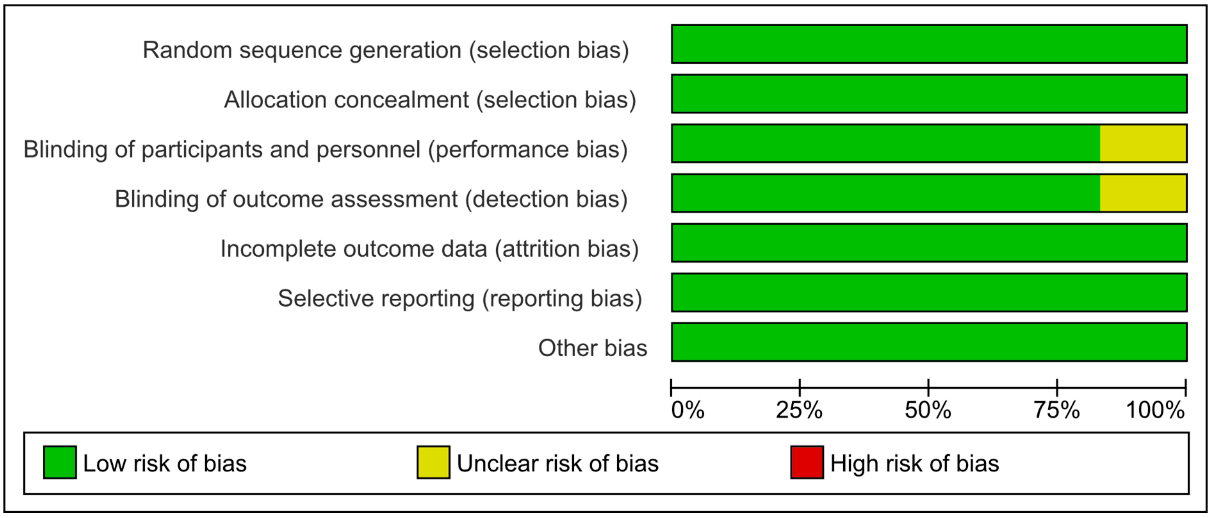

(B)

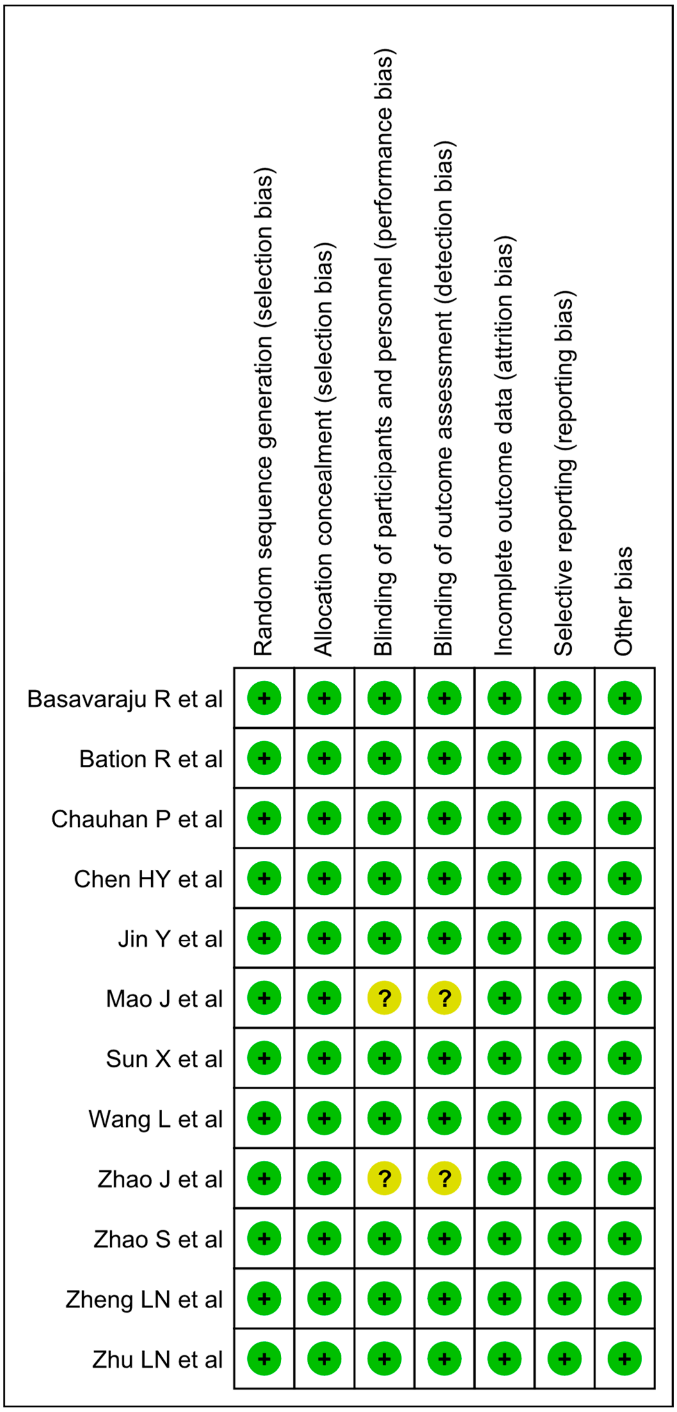

Random sequence generation (selection bias)
Allocation concealment (selection bias)
Blinding of participants and personnel (performance bias)
Blinding of outcome assessment (detection bias)
Incomplete outcome data (attrition bias)
Selective reporting (reporting bias)
Other bias
Basavaraju R et al
Bation R et al
Chauhan P et al
Chen HY et al
Jin Y et al
Mao J et al
Sun X et al
Wang L et al
Zhao J et al
Zhao S et al
Zheng LN et al
Zhu LN et al

8. Supplementary Table S4: GRADE (Grading of Recommendations, Assessment, Development and Evaluations) form

**Initial rate**	**Factors up**	**Evidence**	**Overall rating**
study design: RCT- high quality	risk of bias of individual study limitations	overall low risk for all individual studies	Medium quality
	imprecision	PANSS-N effect size is meaningful compared to clinical significance	
	publication bias	no obvious bias, Egger's test p=0.475	
	dose-response gradient	subgroup analysis of total number of pulses	
	large magnitude of effect	moderate effect size	
	**Factors down**	**Evidence**	
	indirectness of evidence	There are 3 studies on patients with non-dominant negative symptoms	
	inconsistency of results	I^2=0.90	
	inconsistency of results- subgroup analysis and sensitivity analysis	I^2 ranges from 0 to 0.93	
	**Factors no change**	**Evidence**	
	confounding analysis	not applicable for meta-analysis of RCTs	
Summary	Further research was likely to affect the reliability of the efficacy evaluation results		
